# Precarious work on the rise

**DOI:** 10.1186/s12889-024-19363-3

**Published:** 2024-07-31

**Authors:** Melissa Perri, Patricia O’Campo, Paneet Gill, Virginia Gunn, Rachel W Ma, Pearl Buhariwala, Elham Rasoulian, Wayne Lewchuk, Sherry Baron, Theo Bodin, Carles Muntaner

**Affiliations:** 1 Dalla Lana School of Public Health, University of Toronto, Toronto, ON Canada; 2 MAP Centre for Urban Health Solutions, St. Michael’s Hospital, Toronto, ON Canada; 3 Cape Breton University, Halifax, NS Canada; 4 Unit of Occupational Medicine, Institute of Environmental Medicine, Karolinska Institutet, Stockholm, Sweden; 5 Department of Economics and School of Labour Studies, McMaster University, Hamilton, ON Canada; 6 Barry Commoner Center for Health and the Environment, Queens College, City University of New York, New York, Queens USA; 7 Center for Occupational and Environmental Medicine, Stockholm Region, Stockholm, Sweden; 8 Lawrence S. Bloomberg School of Nursing, University of Toronto, Toronto, ON Canada

**Keywords:** Precarious employment, Mental health, COVID-19, Health and social inequalities

## Abstract

Precarious employment (PE) is non-standard employment with uncertain and unstable contract duration, low wages, and limited labour protections and rights. Research has associated PE with workers’ poor mental health and well-being; however, this association has been studied primarily using quantitative methods. This qualitative study seeks to examine the mechanisms between PE and mental health in the context of the COVID-19 pandemic in Ontario, Canada. Specifically, it aims to address: (Benach J, Muntaner C. Precarious employment and health: developing a research agenda. J Epidemiol Community Health. 2007;61(4):276.) How do PE and working conditions impact the mental well-being of workers and members of their close families or households?; and (Kreshpaj B, Orellana C, Burström B, Davis L, Hemmingsson T, Johansson G, et al. What is precarious employment? A systematic review of definitions and operationalizations from quantitative and qualitative studies. Scand J Work Environ Health. 2020;46(3):235–47.) How has the COVID-19 pandemic shaped these relationships? Semi-structured interviews were conducted with a sample of 40 individuals aged 25–55 engaged in PE during the first wave of the COVID-19 pandemic or whose employment was terminated due to the pandemic. Results showed that PE amplified mental health symptoms and illnesses for workers and their families. These experiences were described as chronic, where impacts were exerted on precariously employed workers through systemic discrimination and racism, colonialism, workplace hierarchies, and gendered ideologies. PE negatively impacted mental health through emotional stress about employment and income instability, insecurity, and loss; added pressure for households where both partners are engaged in PE; impacted ability to maintain or improve overall health and well-being; and barriers to social connectedness. Overall, this study characterizes multiple dimensions of PE and the consequences they have on the mental health of workers and their families.

## Introduction

Precarious employment (PE), defined as non-standard employment of limited duration with a lack of financial security, labor protections, and rights, [1–3] has increased globally over the last decades [3, 4]. The COVID-19 pandemic has exacerbated the risks of un- and under-employment, and loss of income for those with precarious employment conditions [5, 6]. There is a growing body of evidence that acknowledges the impact of precarious employment on workers’ health and well-being [7–10]. This sizable, primarily quantitative body of empirical literature has shown associations between employment precariousness and poor mental health [11–18]. Those in PE demonstrate a high incidence of common mental disorders and syndromes such as depression and anxiety, substance use, sleep problems, and suicide attempts [11, 12]. Compared to permanent workers, the probability of experiencing poor mental health is almost doubled in precariously employed workers [13]. Furthermore, it has been shown that higher levels of precariousness are associated with experiencing high stress [14], depression, and anxiety [15].

The probability of having a psychotropic medication prescription is also higher for workers in temporary contracts, those with more days of work under temporary contracts, and those with frequent changes in temporary contracts [16] compared to those with permanent contracts. A systematic review of quantitative and qualitative studies evaluating the mental health status of precarious migrant workers showed that among different dimensions of PE, job insecurity, low income, and disrespectful employer behavior were largely responsible for poor mental health problems in this population [13]. Another systematic review found moderate evidence on the association between poor mental health and job insecurity, however, also brought attention to the few studies that explore this relationship especially amongst precariously employed workers who are often difficult to reach [16]. Employment precarity brought on by COVID-19 may also impact mental health status [19], where women experiencing job precariousness in response to the pandemic have been shown to report greater anxiety than stably employed women [20]. While it’s likely that the ongoing COVID-19 crisis has negatively impacted the mental health and well-being of workers in PE, research in this area remains limited.

The relative dearth of qualitative studies leaves a gap in understanding of the theoretical social mechanisms underlying the relationship between PE conditions and poor mental health. For example, theory suggests that employment insecurity leads to mental health problems through income insecurity, but this has been studied mostly in relation to men [14]. We seek to expand the theoretical understanding of the mechanisms by which PE creates poor mental well- being to address some remaining theoretical gaps. Do workplace structures such as managerial hierarchies play a role in the association between PE and depression [21] through lack of control and helplessness? What role does systemic discrimination play in creating stress and poor mental health among precarious workers? These and other crucial questions about mechanisms by which PE conditions cause mental health problems can be illuminated with qualitative research with or without the addition of quantitative studies. Qualitative research seems to be well-suited for the description of these mechanisms of PE and, consequently, identifying areas of intervention [22].

According to the above considerations, we conducted a single case study of the province of Ontario, Canada during the first wave of the COVID-19 pandemic to examine the mechanisms underlying the association between PE and mental health. Specifically, our research questions were: (1) How do PE and working conditions impact the mental well-being of workers and members of their close families or households?; and (2) How has the COVID-19 pandemic shaped these relationships?

## Methods

This study is part of the Precarious Work Research (PWR) project, a mixed methods study which took place in Sweden, Belgium, Spain, Chile, the United States, and Canada. The aim of the PWR project was to develop a more comprehensive understanding of the mechanisms in which employment and related social policies could support workers and their families. Detailed information about the quantitative and qualitative methods of the PWR project are described elsewhere [23, 24]. Because data collection was initiated during the early phase of the pandemic, this study pivoted to also explore the impact of COVID-19 on employment conditions, to respond to the rapidly changing conditions in which this study commenced. The following analysis focuses specifically on a single site, Ontario, Canada.

## Paradigmatic standpoint

In the context of qualitative data analysis, we understand that a “paradigmatic standpoint” refers to the foundational set of beliefs and assumptions that guide how we, the researchers, view the world and, consequently, how we approach the collection, interpretation, and analyses of data [25]. The paradigmatic standpoint shapes our methodological choices, including how we understand reality (ontology), what we consider valid knowledge (epistemology), and the values that guide their research (axiology) [26]. The assumptions of our paradigmatic standpoint, Scientific Realism [27], are as follows: we assume an objective reality outside the observer (Ontology); some knowledge can be gained about this reality (Epistemology); there are methods to gather and analyze data, that allow us to understand some aspect of the reality (Methodology); there are some objective values linked to factual scientific claims (axiology) [28].

### Data collection

The interview sample used was drawn from participants in a quantitative survey on precarious employment conducted with 451 respondents between October 2020 to June 2021. All survey respondents had experienced precarious employment either at the time of the survey or within the three months preceding it, were not full-time students, were aged 25–55, and their employment was permanently or temporarily impacted/terminated by the COVID-19 pandemic. Using the employment precarity index, the survey asked questions, which measured 10 direct and indirect measures of employment insecurity to identify precarity level, including: employment relationship, income uncertainty, scheduling uncertainty and relationship uncertainty. The final interview sample included workers who were: (1) informally employed (not making pension contributions or paying taxes); (2) had a fixed contract; (3) engaged in part-time employment; and (4) received employment through a gig or temp agency. Given the fact that our sample was aged between 25 and 55, we have minimized representation of those who identify as students or those who are in or approaching retirement. This study gained ethics approval through the St. Michael’s Research Ethics Board (REB 20–110, MAP Centre for Urban Health Solutions, Li Ka Shing Knowledge Institute, Unity Health, Toronto, ON, Canada). Of the above-described sample, 40 individuals were selected based on their Employment Precariousness Index scores (higher > 16 or lower precarity ≤ 15, gender, age, and geographical location within Ontario) for 1-hour semi-structured interviews. Interviews were conducted by four research team members who were trained interviewers (PO, VG, ER, and, PB). Interview questions asked respondents about their employment relationships, impacts of employment situation on the health of the participant and their family members as well as policy impacts (both existing and non-existent policy) on the health and well-being of respondents and their families. We allowed participants to adopt and discuss their own conceptualizations, perceptions and experiences of physical and mental health and did not provide a fixed definition for them to follow. All questions were asked in the context of the COVID-19 pandemic. All interviews were conducted virtually using Zoom Healthcare video or audio conferences, to ensure COVID-19 distancing precautions. Interviews were transcribed using Otter AI software (otter.ai). Eight coders (PO, VG, PB, ER, MP, MA, SS, SZ) reviewed and corrected each transcript.

### Data analysis

The coding framework for this study was developed with insight from each country and site participating in the project, with the goal of harmonizing categories of findings across the six country locations. Thematic analysis was used with small adaptations being made to account for site specific contexts. NVivo 12 was used to code all transcripts by eight members of the research team in an iterative and collaborative manner (PO, VG, PB, ER, MA, MP, SS, SZ) for the analyses across the six countries [29]. For this analysis of employment insecurities experienced in Canada, a second, more detailed coding and thematic analysis was conducted to answer the research question for this paper and was conducted by PO, CM, MP, and VG. The full team reviewed these findings and provided feedback accordingly.

## Results

We had a diverse sample of 40 participants. 55% identified as women, and 7.5% reported being gender non-binary. Almost half were 34 years of age or younger. 32% were born outside of Canada and 57% had household incomes of $2400 or less per month. Forth-2% reported having a physical or mental disability. The majority were from Toronto, 65%, and 17% were from Northern Ontario and 12% were from Southern Ontario. Experiencing PE influenced not only participants’ mental health and well-being, but also that of their families in a variety of ways. Given the fact that our interviews took place during the pandemic, it was almost impossible to separate the specific impacts of COVID-19, which permeated most answers. Participants shared how multi-faceted aspects of PE were experienced by them, including employment insecurity, income insecurity, benefits insecurity, and rights-based insecurities. The ongoing reflections reveal how participants faced multiple forms of precarity at various time points, leading to heightened mental health issues for workers and their families. The mental health implications PE had on workers ranged in presentation and significance including mental distress, anxiety, depression and even suicidal ideation. These experiences were exacerbated by insecurities around duration and risk of loss of employment, schedule uncertainty, income insecurity and lack of employer-based benefit and were described by participants as chronic. To our surprise, level of precarity was not associated with changes in mental health outcomes relating to PE.

Participants reported myriad pathways by which precarious employment and working conditions impacted their mental well-being. We have presented them in four sections below.

### Worries about employment and income instability, insecurity, and loss

Emotional stress was a common mental health implication identified by participants, resulting from the conditions of PE. Stress was driven by factors such as the instability of income sources, scattered paychecks, constant job hunting, and multiple job losses. The COVID-19 pandemic exacerbated these negative impacts. For instance, a young man experiencing high employment precarity expressed how he has had stress over finding a job after his current contract ended and how COVID-19 intensified this situation:

*Yeah*,* because even while I was working on the contract*,* I was always thinking about*,* you know*,* where am I going to next find work. […] So I would say it was just a stressful time all around. And I’d say that the pandemic mostly has made it difficult for me to find*,* like harder than it ordinarily would have been*,* to find work. I think that since I lost my job before the pandemic*,* I think that that would have happened regardless*,* but like*,* but I think the job search has been harder*,* because there’s been a lot of people who’ve actually lost their jobs because of the pandemic. And it means that there’s more people to compete with. And there’s actually just fewer positions available*,* because otherwise those people wouldn’t have lost their jobs.* [Younger gender variant, high precarity, policy analyst]

The employment uncertainty associated with PE was also described to influence participants’ stress and well-being. As an example, a respondent who worked as a teaching assistant explained that her contract was terminating soon, which has increased her stress. In addition, stress was augmented due to inability of budget planning and not knowing when or where the next paycheck will come from:

*Um*,* well*,* I mentioned earlier that my contract [is] going to be ending up soon. And I mentioned about the inability to like kind of budget long term. So that’s one method of stress. […] And more so than previous times of my life when I’ve had like a contract ending or something like that*,* where I’ve been like*,* Well*,* I know I can like*,* I can find something*,* even if it’s not even from under employed until be able to*,* like*,* pay the bills*,* well*,* if I have something else. But right now*,* once my contract ends*,* I don’t know when my next like*,* paycheck would be coming in. So that’s obviously a big source of stress.* [Younger woman, low precarity, teaching assistant]

Another respondent with high precarity as a contract lecturer explains that he faced depression and anxiety after losing his job:

*When I lost my job*,* I definitely felt anxiety*,* and I definitely felt depression*,* and the anxiety was primarily about*,* okay*,* I realize I had a little bit of money saved*,* I did get a severance that tied me over. […] I got a severance pay that made a pretty big difference tiding me over for a while. Notwithstanding that severance [referring to payment]*,* I still had a lot of anxiety about what was going to happen when it ran out. […] Um*,* so it*,* I would say it affected me very negatively. Anxiety and depression over the uncertainty.* [Older male, high precarity, contract professor]

Many participants explained that they experienced anxiety and stress due to an exposure to unstable and unpredictable work schedules that relate closely to income insecurity. This respondent mentioned how experiences of income instability caused high volumes of stress:

*It’s stressful. Because*,* like*,* as I said at the beginning*,* like*,* there is no guaranteed income all the time. So I*,* I can’t say that*,* Oh*,* well*,* yeah*,* I made X amount of dollars each*,* you know*,* week or month. It’s like*,* it could be anything so that*,* you know*,* that’s kind of stressful. That’s*,* that’s the main part of it. That’s the biggest kind of stress. Yeah.* [Younger woman, high precarity, job title unknown]

Income insecurity due to PE caused stress over meeting basic needs such as food, housing, clothing, and education. This stress intensified during the COVID-19 pandemic, as this respondent with high precarity expressed:

*My well-being is just you know what I have to worry about now before I’ve didn’t have that to worry about where my next meal was gonna come. But now I’m worrying about my meal*,* the rent. So all this is adding more stress. Psychologically.* [Older woman, high precarity, government contract worker]

### Added pressure when both partners in a household are engaged in precarious employment

Participants described the added stress faced by family members when multiple individuals were precariously employed. Some participants described hiding instances of precarity to avoid adding to stress faced by partners, children, or other family members:

*You know*,* and that really affected the household. Because I never realized that he had been hiding that from me for a couple of months. Because he didn’t want to stress me out. You know*,* because he was the one basically making all the money at the time*,* right? When*,* yeah*,* so he wanted to protect me*,* but you know*,* coming home every night*,* he was also working midnight shifts. So his body and brain were just like*,* I don’t know what time it is. I don’t know anything. Right. So that was really hurt on us as a couple because I felt*,* you know*,* kind of like*,* why did you lie to me*,* but I understood. So*,* you know*,* that had an impact on our relationship.* [Younger woman, high precarity, job title unknown]

### Impacts on ability to maintain or improve overall health and well-being

As discussed above, for some participants, the nature of their PE and related income and employment insecurity, in addition to the limitations put forth by COVID-19, negatively impacted their ability to maintain or improve their overall mental health and well-being. Being involved with PE was described to influence this individual’s aspirations for the future and sense of security for themselves and their families which in turn, led to feelings of discontent and sadness:

*Um*,* I have a partner who I’ve been with for two years*,* we don’t live together. But I believe that one of the reasons that that I don’t have a family is because of the amount of time I’ve spent without… a… I’m always employed*,* but the work that I do is always changing and it makes it hard to settle in one place and*,* and build a life. Not to mention build capital just like build equity and savings.* [Younger male, low precarity, contract professor]

Another factor that affects the mental health of individuals engaged in PE is the way in which they perceive employers to value their work. Feeling threatened by employers regarding the ease of worker replacement was explained as negatively affecting individuals’ mental health. As one individual stated:

*Well*,* yes*,* it does. It affects my mental health quite significantly*,* I feel that they are very*,* they are impatient to get their work done. And they have told me that I’m quite replaceable*,* which is unfortunate. I don’t feel like I have a lot of security there. I have to work very hard for the pay I make. And I feel like it’s a very bad long-term plan.* [Older male, high precarity, driver]

However, some workers who switched to remote work during the pandemic benefited from more flexible working hours, which allowed them to incorporate more active breaks such as going for walks.

*Yeah*,* well*,* I’m just Now that everything’s remote*,* you know*,* it’s nice when you can tune into an exercise program. On your lunch break*,* belt*,* where I’ve worked*,* I’ve always had the chance to go for a walk*,* or*,* you know*,* just take a different type of break*,* you know*,* […] Where*,* you know*,* if you’re rushing to work*,* and you have a full work week*,* you might not get those chances and opportunities to enjoy your surroundings in your environment. And it’s just made me have better decisions*. [Older woman, high precarity, job title unknown]

Our findings confirmed that, in addition to the direct impacts on non-standard workers’ own stress level, PE also affected the stress levels of family members. Numerous workers in our sample talked at length about the ongoing stress experienced by members of their close and extended family in response to their [workers’] PE. Our participants shared touching examples of life partners, children, siblings, parents, and even more distant relatives being stressed and worried about their employment not being secured, predictable, or financially adequate.

*I’m the daughter of an immigrant. So*,* this type of labor is not understood at all*,* which causes a lot of rifts between the two of us*,* especially given my educational background. […] And this type of what we call skunk work*,* essentially like that underground that nobody sees. Some stuff like*,* especially for my mother*,* like she does not understand how this happens*,* and she does not understand how this helps me. So I think there’s there is often a huge defense*,* I have to constantly defend my labor choice*,* which is not really a way like I yes*,* I am freely choosing this*,* but also only to the options that are available to me during this time. So that’s been very tough*,* because of like the*,* the lack of consistent hours*,* and the very*,* very*,* very low wages that I received from this*,* I do have the soul very much rely on my mother to be able to provide for me*,* and*,* you know*,* keep our housing situation*,* which is I certainly I think a strain because at this age and being female*,* she was expecting my lifetime to be married with a partner*,* beginning my own family. So that added stress with this type of labor. It’s certainly been a cocktail for like*,* a lot of stress.”* [Younger woman, high precarity, personal assistant].

### Impact of precarious employment on social connectedness

In addition to the role PE played in facilitating or inhibiting health related behaviours, participants also spoke to its impact on social connectedness and mental health and well-being. Many explained that having multiple jobs, in addition to unique employment related demands (e.g., handling difficult cases as a social worker, being on one’s feet during long and irregular working hours), created challenges in maintaining social connections both inside and outside of work environments:

*Um*,* I would say the hardest thing is like*,* sometimes I just don’t really want to talk to anybody. And I don’t think sometimes people understand that either. […] And there’s some days where I’m just I’m so far beyond what I can do for the day in terms of like*,* just mental and emotional capacity*,* that I just like*,* I feel like I can’t be present in any sort of*,* like any*,* any aspect of my social life […]. So I think sometimes it affects how present I can be. And then also*,* just like how like good of a of a friend or a daughter or a sister is I just feel like sometimes I can like closet myself into a bit of like an introverted bubble where I don’t really want to talk to anybody.* [Younger woman, low precarity, bartender and social worker]

These feelings of disconnection were augmented significantly during COVID-19 due to the shift to remote working. Participants described feeling isolated lonely, depressed, and lacked the social stimulation which came with going into work every day:

*The virtual barrier really did change the way that*,* that kind of developed in that*,* you know*,* there was no opportunities to really form a personal relationship with anybody else*,* right? Like it*,* you know*,* you’re in a meeting or virtual meeting*,* that’s basically it. If anything*,* you send a couple of messages between one another*,* that’s it*,* right. So I would say*,* if it affected me at all*,* it would be in that it*,* there was no real room to form any sort of connection or relationship*,* which is something that in my past jobs and my past positions has been quite rewarding in many ways.* [Younger male, high precarity, policy analyst]

For some participants, mental health issues triggered during the pandemic were linked to the overall uncertainty and change in daily routines:

*Yeah*,* I would definitely say we were we were*,* we were at each other’s throats a little more than we would have been […] you don’t see an end in sight*,* you start to kind of nitpick at each other and get frustrated over the little things for sure. And you’re not seeing friends and you’re not going and doing your normal fun activities*. [Younger woman, high precarity, receptionist]

For others, it was the social isolation associated with changes in the provision of in-person schooling or childcare services, which created important implications for family dynamics and caregiving responsibilities. These changes augmented stress, anxiety, and discontent:

*And I’m not efficient in this world anymore. And you know*,* my kids kind of took a backseat to all of that. And so did my parents took a backseat to a lot of that because it was a heck of a lot of responsibility that none of us knew how to do. None of us*,* because nobody’s ever done this before. So it’s not like there was a guidebook of how to do this. So it was just a lot of stress that my kids definitely felt. And by the end of the day*,* I was so exhausted with my 12 people’s jobs that no*,* we’re not going to the corner store. No*,* we’re not going for a walk. No*,* we’re not going to the park*,* I’m having a nap. And just COVID itself is exhausting*,* right. So*,* it was very*,* very trying time.* [Younger woman, low precarity, employment counselor]

The added pressures of working from home while the kids were not in school created high volumes of stress and disconnection:

*So*,* I still needed an hour or two to decompress before I even really got to real family time. […] So*,* you know*,* they only really got me for a couple hours a day*,* really. So*,* they did they took a little bit of a kicking over the summer because of that. And again*,* I’m home. So why aren’t we going camping? Why aren’t we out at the beach? Because I’m working and then a little bit more snappy on them than they deserve. […] But wellness*,* I think kind of definitely took a little bit of a kick just because of how much I had to devote to work that I didn’t have to do before they weren’t used to me having to be so attached to my employment that I didn’t have time for that just didn’t work.* [Younger woman, low precarity, employment counselor]

The constant navigating of more than one job to make ends meet, the unpredictable schedules, or long hours limited workers’ opportunities to plan for and spend time with their families. This was described by participants as heavily impacting family dynamics and in turn, causing even more stress for both individuals and their families:

*Yeah*,* it’s*,* um*,* I think there is an awareness that I don’t have as much time for the family as they would like*,* you know*,* I don’t have*,* I don’t have a lot of time to spend with my kids or my husband. […] You know*,* so I think it is stressful for them. And you know*,* plus the fact that you know*,* every*,* everyone is kind of having to do more*,* it’s everyone’s having to work harder. Everyone’s everyone*,* no one has as much time as they used to have. You know*,* I used to take on a lot are not able to take on everything that I used to*,* and some of that load falls on the rest of the family*,* and yet everyone is stressed. Yeah* [Older woman, high precarity, self-employed].

Admittedly, COVID-19 related mental health issues are not unique to non-standard, precarious workers, however, given that they are often lacking access to sufficient financial resources and/or additional supports, they could be affected to a higher degree by such problems.

## Discussion

To date, few studies have assessed the relationship of PE working conditions on the mental health of workers and their families, the mechanisms underlying those relationships, and the role that COVID-19 pandemic plays in potential negative outcomes. This study aimed to fill this knowledge gap. We found that engagement in PE, particularly during the COVID-19 pandemic, led to a variety of symptoms and experiences of poor mental health of workers and at times, of their families. Specific outcomes mentioned by the participants in this analysis included increased stress and concern surrounding employment and income instability, insecurity, and loss, particularly when multiple individuals in a household experience PE; increased anxiety and depression; and a negative impact on the ability of workers to remain socially connected.

An important aspect of our findings is how interrelated COVID-19 was in the above noted outcomes given its role in facilitating experiences of isolation, lockdown, and severe income and employment loss. The relevance of COVID-19 in augmenting negative mental health outcomes among individuals engaged in PE is particularly relevant in Ontario, Canada, given the uniquely long lockdown periods (i.e., over 2 years) and provincial wide economic instability [30]. Our findings are aligned with existing quantitative research that explores the relationship between PE and mental health outcomes. Jonsson and colleagues (2021) conducted a cross-sectional study with 401 individuals engaged in PE in Sweden and demonstrated an association between PE and poor mental health outcomes [31]. This study demonstrated how degree of precariousness, ranging from low precariousness to high precariousness, influenced the magnitude of self-rated poor mental health status [31]. Similarly, Matilla-Santander and associates (2021) demonstrated a strong association between engagement in PE, level of precarity, and outcomes relating to anxiety in their cross-sectional study based on the 6th European Working Conditions Survey [32].

We should note a few limitations of this study. First, we had to rely on precarious workers who spoke English as a first or second language and therefore we missed individuals who are in Canada but do not speak English. We may be missing an important group from this study. Second, we asked questions about the impacts of the COVID-19 pandemic early on in the global public health emergency. We may have missed longer term impacts of the pandemic that could not have been reported on at the time that we did the interviews in 2020–2021. This study has a variety of strengths. Namely, it is one of the first studies to qualitatively explore the relationship between PE and mental health outcomes for workers and their families. We include here a robust sample of the perspectives of those living with PE, highlighting a range of experiences of workers facing higher and lower precarity. We also ensured that the sample included in this study was diverse demographically and geographically within Ontario. One limitation is that these perspectives are of those who resided within Ontario, limiting insight into how different geographic settings and structures may influence PE and related outcomes.

Currently, limited research explores how the varying dimensions of PE (e.g., systemic discrimination and racism, colonialism, gendered ideologies, and power dynamics) influences the health and well-being of workers and their families. As highlighted in our findings, and supported by the theory that informed this study [4, 18, 22, 33], workplace and societal structures, systemic discrimination and racism, and gendered ideologies all influence the mental health of workers. Further, we show how the COVID-19 pandemic has facilitated mechanisms which lead to PE and negative health outcomes for workers (e.g., pay equity, employment security, provincial economic stability). Future work must continue to examine what the diverse mechanisms of PE are, how they influence the health and well-being of workers, and more importantly work to eliminate the societal conditions which create PE and associated poor mental health.

## Conclusion

This study highlights the role PE employment plays in influencing the mental health of workers and their families. We demonstrate how engagement in PE manifests into experiences of high stress, trauma, anxiety, and depression for not only workers but also their families. Further, we provide unique insight into how the COVID-19 pandemic furthered negative consequences of PE. Considering the complexities of PE is essential in minimizing the multi-level forms of harm faced by workers and their families. We urge future scholars to explore further mechanisms of PE which produce negative outcomes for workers and their families and work towards eliminating PE conditions altogether.

## Data Availability

Data were collected in a manner that must maintain confidentiality of the participants. Data are available from the corresponding author upon reasonable request. Data will be shared in aggregate or anonymised form due to privacy concerns upon reasonable request.
